# Antioxidative and Antiapoptotic Effects of Delta-Opioid Peptide [D-Ala^2^, D-Leu^5^] Enkephalin on Spinal Cord Ischemia-Reperfusion Injury in Rabbits

**DOI:** 10.3389/fnins.2017.00603

**Published:** 2017-10-31

**Authors:** Danyun Fu, Haitong Liu, Shitong Li, Lianhua Chen, Junyan Yao

**Affiliations:** Department of Anesthesiology, Shanghai General Hospital, Shanghai Jiao Tong University School of Medicine, Shanghai, China

**Keywords:** DADLE, spinal cord, ischemia-reperfusion injury, oxidative stress, apoptosis, rabbit models

## Abstract

**Background:** In our previous study, we found that regional administration of delta-opioid peptide [D-Ala^2^, D-Leu^5^] enkephalin (DADLE) could provide dose-dependent protection on spinal cord ischemia-reperfusion (I/R) injury in rabbits. However, the relative protective molecular mechanisms underlying this neuroprotection remain unclear. The purpose of this study was to investigate whether DADLE provided the protection in spinal cord I/R injury through its antioxidant property by decreasing malondialdehyde (MDA) and nitric oxide (NO) levels and increasing glutathione peroxidase (GSH-Px) and superoxide dismutase (SOD) levels and through its antiapoptotic capacity by inhibiting caspase-3 and p53 expression.

**Methods:** The rabbits were divided into three groups. The animals in Group NS and Group DADLE were administered with normal saline (NS) or DADLE via aorta during 30 min of ischemia respectively, while the one in Group Sham received no intervention. During the period of reperfusion, the rabbit's blood samples were collected for enzyme-linked immunoabsorbent assay (ELISA) examinations of MDA, NO, GSH-Px and SOD. At 48 h after reperfusion, the lumbar spinal cords were harvested for immunohistochemical, real-time polymerase chain reaction (PCR) and western blot studies to detect the caspase-3 and p53 expressions.

**Results:** The activities of serum MDA and NO showed significant reductions in the DADLE group as compared with the control group. By contrast, the levels of serum GSH-Px and SOD were significantly higher in the DADLE group than those in the NS group. In addition, caspase-3 and p53 expression were significantly increased in the NS group, while DADLE mitigated these changes.

**Conclusions:** The protective effects of DADLE at the dosage of 0.05 mg/kg may be related to its antioxidant and antiapoptosis properties in the rabbit model of spinal cord I/R injury.

## Introduction

Spinal cord ischemia-reperfusion (I/R) is one of the most serious complication of thoracoabdominal aortic surgery, leading to postoperative paraplegia in 4 to 16% of patients (Zvara, 2002). Despite some efforts to develop neuroprotective agents in experimental spinal cord ischemia, few so far has been translated into clinical application. In our previous research, we revealed that the aorta-infused [D-Ala^2^, D-Leu^5^] enkephalin (DADLE), a synthetic delta-opioid peptide, could approximately reduce the rate of paraplegia by more than 50% and increase the surviving neurons number by 3 times in a rabbit model of spinal cord I/R (Liu et al., 2015a). In order to achieve more reasonable utilization of agent, it is essential to investigate the mechanisms underlying neuroprotective effects of DADLE in the spinal cord.

Oxidative stress reaction is now believed to play a major role in the progression of several organ I/R injury such as brain (Liu et al., 2015b), liver (Liu et al., 2014), and kidney (Nie et al., 2016), including spinal cord (Wang et al., 2017). During I/R, the oxidative processes resulting from the abundant free radicals may cause protein breakdown, lipid peroxidation and DNA damage. The endogenous antioxidant system alleviates these damages through enzymatic substances, such as glutathione peroxidase (GSH-Px) and superoxide dismutase (SOD). In addition, several authors have demonstrated that the neuroprotective effects of various antioxidative therapies, for example, melatonin and oxytetracycline (Topsakal et al., 2003) were regarded as promising targets for therapeutic interventions in spinal cord I/R injury. DADLE has been demonstrated to promote the survival of neurons in the central nervous system including decreasing methamphetamine-induced dopaminergic transporter loss through suppressing reactive oxygen species formation and inhibiting brain lipid peroxidation (Tsao et al., 1998). However, it remains unknown whether DADLE can protect the I/R-induced spinal cords injury through antioxidant property.

Moreover, neuronal apoptosis induced by I/R damage has been reported as another mechanism for neurological deficits in the spinal cords (Jia et al., 2012). Spinal cord I/R injury causes disorders of energy metabolism and produces over-produced free radicals, which exacerbate mitochondrial membrane permeabilization and lead to activation of caspase-9 and caspase-3, subsequently trigger apoptosis (Hayashi et al., 1998). Thus, inhibition of apoptosis through pharmacological pathway might be a potent neuroprotective strategy. DADLE was shown to preserve serum-deprived rat pheochromocytoma cells through an antiapoptotic property (Hayashi et al., 2002). We therefore assessed the effects of DADLE on the neuronal apoptosis in the spinal cords.

The objective of the current study was to investigate the antioxidative effect of DADLE based on the detection of malondialdehyde (MDA), nitric oxide (NO), GSH-Px and SOD levels. Meanwhile, we also studied the antiapoptotic effect of DADLE by evaluating the expression of caspase-3 and p53. We hypothesized that DADLE provided the neuroprotection of spinal cords through its antioxidant activity by decreasing MDA and NO levels and increasing GSH-Px and SOD levels, and through its antiapoptosis property associated with caspase-3 and p53.

## Methods and materials

### Animals preparation

The experiment and animal care protocol were approved by the Animal Care and Use Committee of Shanghai Jiao Tong University (Shanghai, P.R. China), and complied with the “Guide for the Care and Use of Laboratory Animals” published by National Institutes of Health (National Academy Press, Washington DC, revised 1996). Twenty-two New Zealand white rabbits (male and female) weighing 2.5 to 3.0 kg were randomly allocated to one of the following three groups: Group NS (*n* = 8), Group DADLE (*n* = 8) and Group Sham (*n* = 6). Spinal cord ischemia was induced by aortic clamping at infrarenal aorta. During the ischemic period, the rabbits in Group NS and Group DADLE received normal saline or DADLE 0.05 mg/kg through the clamped aorta, while the animals in Group Sham underwent the same surgical schedule but without aortic clamping.

### Induction of spinal cord I/R injury

Animals were anesthetized by the intramuscular administration of ketamine (25 mg/kg) and atropine (0.10 mg/kg). The right ear artery was catheterized for arterial blood pressure and heart rate monitoring and blood specimens sampling. Then they were given endotracheal intubation and mechanical ventilation according to the following parameters: tidal volume, 8 mL/kg; respiratory rate, 30 bpm; ratio of inspiratory time to expiratory time, 1: 1.5; and fraction of inspired oxygen, 1.0. General anesthesia was maintained by intravenous injection of midazolam 0.5 mg/kg, fentanyl 8–10 μg/kg, and vecuronium 0.2–0.25 mg/kg in interval. Core temperature was maintained between 37–38°C supported by a heating lamp throughout the procedure.

The model of spinal cord ischemia was established as described previously (Liu et al., 2016). In brief, an incision was made in the right iliac region and the right femoral artery was exposed. Sequentially, an abdominal incision was made and the retroperitoneal infrarenal aorta was exposed. At the time after heparinization for 5 min, a 22-gauge PA catheter was inserted from the right femoral artery incision to the descending aorta, which was used for monitoring the distal artery pressure and drug perfusion. The tip was advanced approximately 13–14 cm from the site of insertion, and located 1.5 cm distal to the left renal artery. Then aortic cross-clamping was performed by two artery clips, just inferior to left renal artery for 30 min. DADLE 0.05 mg/kg or NS at the same volume was administered into the clamped aortic segments respectively, at the same rate of 3 ml/kg/h during the ischemic period. Meanwhile, the bilateral common iliac arteries were clamped to increase the local drug concentration. The efficacy of the clamping was confirmed by the immediate and continual absence of pulsation and a rapid decrease to less than 20 mmHg in distal abdominal aortic pressure. Immediately at the end of 30 min ischemia, drug infusion was stopped and the artery clips were removed. Then the catheter was removed and the incision were sutured layer-by-layer. The animals were tracheal extubated after they regained consciousness. In order to prevent infection, the animals were injected benzylpenicillin (300,000 U/kg) intramuscularly postoperation. Crede's maneuver was carried out on rabbits to avoid urinary retention three times a day.

### Tissue sampling

At the onset after reperfusion (T1), 1 h after reperfusion (T2), 6 h after reperfusion (T3), 24 h after reperfusion (T4) and 48 h after reperfusion (T5), arterial blood samples from the NS and DADLE groups were obtained for MDA, NO, GSH-Px and SOD analyses. However, the blood samples of the Sham group were just collected at 48 h after the sham operation because we did not perform the aortic clamping in this group. Following centrifugation of 3,000 rpm for 15 min, the supernatant was collected and stored at −80°C in refrigerator until measurement. After completing neurologic deficit score, the spinal cords segments of L4 and L5 were quickly harvested under the general anesthesia and the animals were sacrificed by intravenous injection of sodium pentobarbital (200 mg/kg). One part of the tissue sample was fixed in 10% formalin for 48 h, embedded in paraffin and cut into coronal 4 μm sections for caspase-3 immunohistochemistry examination. Another part of the sample was frozen using liquid nitrogen and then transferred to −80°C freezer for p53 mRNA and protein expression analysis.

### Biochemical analysis of oxidative stress markers

To ascertain the effect of DADLE on antioxidant mechanism concerning spinal cord I/R injury, we measured the contents of serum MDA and NO, two markers of oxidative stress and the activities of GSH-Px and SOD, the most important enzymes to protect against oxidative stress.

#### MDA activity

The level of serum MDA concentration was measured by the thiobarbituric acid method using an MDA kit (Nanjing Jiancheng Bioengineering Institute) according to the manufacturer's instructions. A total of 100 μL of serum sample was mixed with thiobarbituric acid, acetic acid and sodium dodecyl sulphate and incubated at 95°C for 40 min. After cooling with water, the sample was centrifuged at a rate of 4,000 rpm for 10 min. The absorbance values in all tubes were measured at the wavelength of 532 nm using a spectrophotometer. Concentrations of MDA were determined using a standard curve calculated from tetraethoxypropane. The content of MDA was expressed as nmol/L serum.

#### NO activity

To determine the NO activity in serum, nitrite and nitrate were measured as indexes of NO production using an assay kit (Jiancheng Bioengineering Institute, China). In brief, the sample was mixed with the Griess reagent mixture in a 96-well microtiter plate at room temperature for 10 min (Cortas and Wakid, 1990). Nitrite products in the samples were calculated by measuring absorbance at 540 nm with a standard curve established sodium nitrite. The results were expressed as μmol/L serum.

#### GSH-Px activity

The GSH-Px activity was estimated using an GSH-Px kit according to the manufacturer's instructions (Jiancheng Bioengineering Institute, China). In brief, the supernatant was mixed with phosphate buffer, sodium azide, glutathione reductase enzyme, NADPH and H_2_O_2_. The absorbance reduction of NADPH caused by enzymatic reaction at 340 nm were measured after initiation of the reaction. One GSH-Px unit was defined as the enzyme activity required conversion of NADPH to NADP^+^. The GSH-Px activity was expressed as units per mg protein.

#### SOD activity

The serum SOD activity was measured using a SOD kit (Nanjing Jiancheng Bioengineering Institute). It used a xanthine-xanthine oxidase system as a superoxide generator to conduct the inhibition of nitroblue tetrazolium reduction. In brief, 100 μL sample was mixed with the xanthine and xanthine oxidase in potassium phosphate buffer and incubated at 37°C for 20 min. The enzymatic reaction was stopped by CuCl_2_. SOD activity was measured by detecting the absorbance at the wavelength of 450 nm. One unit of activity was defined as the enzyme that caused 50% inhibition rate. SOD activity was expressed as units per mL serum.

### Caspase-3 immunohistochemistry

Caspase-3 immunohistochemistry was processed according to the protocol provided by the manufacturer (Boster Biological Technology co. Ltd., Wuhan, China). In brief, spinal cords sections were deparaffinaged and rehydrated in ethanol and then radiated in sodium citrate buffer (pH 6.0) by microwave for 15 min to achieve antigen retrival. The sections were then incubated with 3% H_2_O_2_ to inactivate endogenous peroxidases for 10 min, following a rinse with phosphate-buffered saline for three times. Subsequently, these sections were incubated at 37°C for 2 h with an anti-caspase-3 primary antibody (1:100, Abcam, Cambridge, MA, USA) and then rinsed in phosphate-buffered saline three times. Next, the sections were blocked with goat anti-rabbit biotinylated secondary antibody (Boster Biological Technology co. Ltd., Wuhan, China) at 37°C for 30 min. After three additional washes in phosphate-buffered saline, caspase-3 signals were visualized by the 3,3′-diaminobenzidine tetrahydrochloride method. Finally, the sections were counterstained with hematoxyline and coverslipped.

Immunohistochemical images were taken with an optical microscope (Zeiss, AxioCam MRc, German). Cells with a blue color were regarded as neurons, while neurons presenting brown color were regarded as caspase-3 positive cells. The normal and caspase-3 positive neurons were counted by two researchers blinded to the experimental assignment. At least three high power fields were randomly selected on each section. The ratio of caspase-3 positive cells in each high power field was used for the statistical analyses.

### Real-time polymerase chain reaction (PCR) analysis of p53

Apoptosis was postulated to participate in neuron injury induced by I/R (Hayashi et al., 1998). To investigate whether DADLE modulated the process of apoptosis in ischemic spinal cords, we assayed the p53 mRNA expression, a reliable index for apoptosis. Total RNA was extracted from the spinal cord tissues using a Trizol reagent (Invitrogen, Carlsbad, CA, USA). Reverse transcription was carried out according to the manufacturer's instructions of the RNA real-time PCR kit (BioTNT Co., Ltd, Shanghai, China). The PCR reaction was performed by adding 2 μL cDNA template, 8.5 μL ddH_2_O, 12.5 μL SYBR Green mix and 1 μL one of the following primers: p53, 5′-CCCGACAGCCAGGTCATC-3′ (forward), 5′-GTTGAAGGTGGTCTCGTGG-3′ (reverse); β-actin, 5′-TGGAGGAGTCGCAGTCGGA-3′ (forward), 5′-GAGGTGGTCAGCAGGTTGT-3′ (reverse). The primers were designed using the Primer5 program and synthesized from Invitrogen (Carlsbad, CA, USA). All samples were tested in triplicate. The real-time PCR was performed with an initial denaturation step at 95°C for 15 min, and a amplification step at 95°C for 15 s and 60°C for 30 s for 40 cycles. Real-time PCR products were separated with electrophoresis and visualized under ultraviolet light after ethidium bromide staining. The relative level of p53 mRNA was calculated by 2^−ΔΔCT^ method. mRNA expression values were normalized using β-actin as an internal control.

### Western blot analysis of p53

In order to further confirm the effect of DADLE on p53, western blot analysis was also performed. Hundred micro gram protein samples underwent 10% SDS polyacrylamide gels and transferred to nitrocellulose membranes. Membranes were blocked with 5% dry milk Tris-buffer and incubated overnight at 4°C with a monoclonal antibody to p53 (1:500; GeneTex, USA). After a rinse with Tris-buffered saline with Tween for three times, the corresponding secondary antibody (Boster Biotechnology Co., Ltd., Wuhan, China) was added and incubated for 1 h. The blots were detected by a chemiluminescence detection system. β-actin was used as an internal reference to correct the variations of different samples. The gray values of the target bands were quantified using an image analysis software (Image Pro Plus 6.0).

### Statistical analysis

All data were expressed as mean ± standard deviation. SPSS 17.0 statistical software package was used for statistical analysis (version 13.0, SPSS Inc., Chicago, IL, USA). The difference in means between two groups was assessed by the Student's *t*-test. Differences in means among three groups were analyzed using one-way ANOVAs followed by intergroup comparison using the Dunnett test. The difference between each time point in different groups was analyzed using two-way ANOVAs with time and drug treatment considered as factors followed by the Bonferroni *post hoc* test. A *P*-value of < 0.05 was considered statistically significant.

## Results

### Assessment of the level of serum MDA

**Figure 1:**
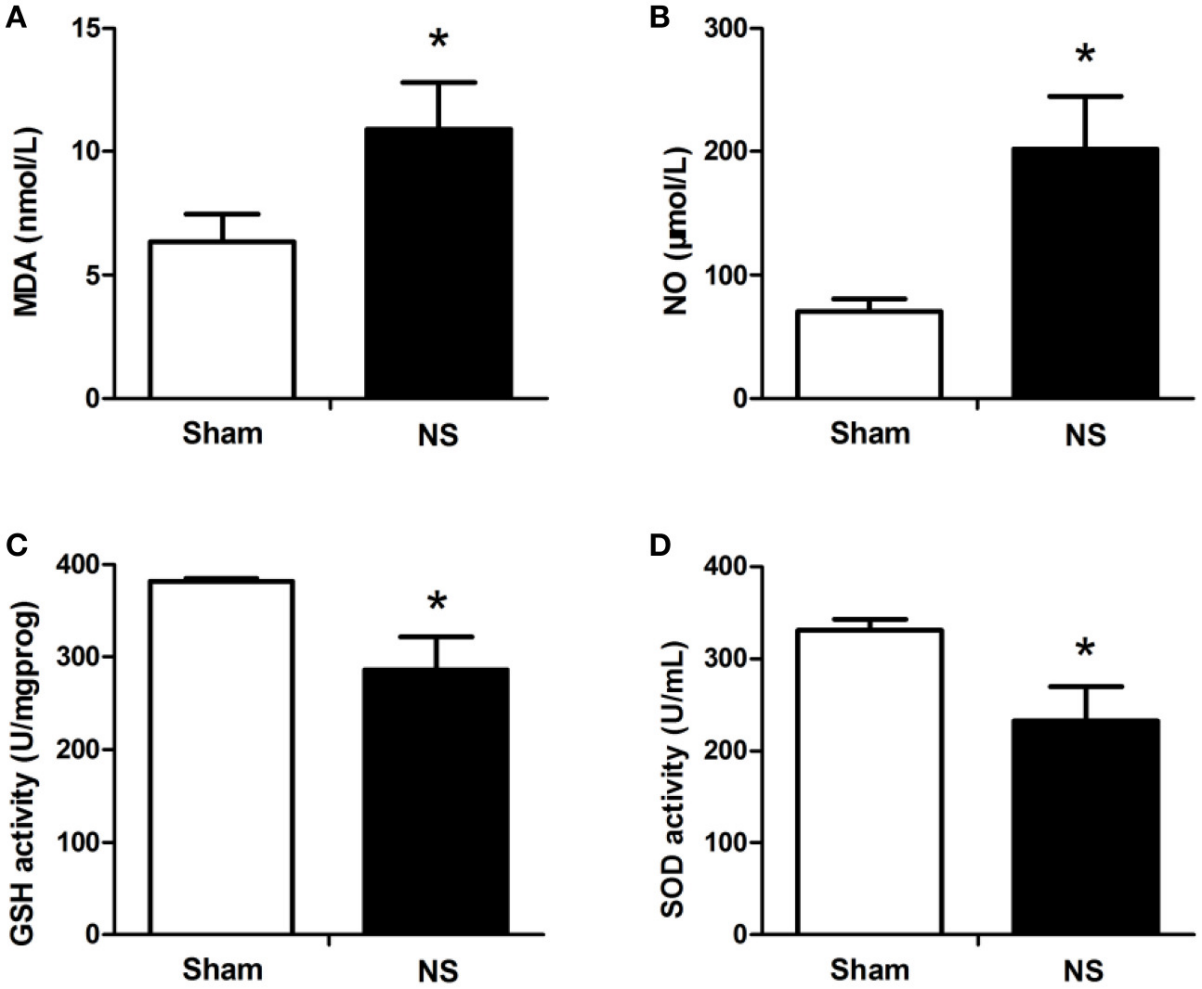
ELISA results of MDA, NO, GSH-Px. and SOD expression in the spinal cords between the NS and Sham groups. Compared with the Sham group, I/R injury significantly increased the levels of MDA **(A)** and NO **(B)** and decreased the levels of GSH-Px **(C)** and SOD **(D)**. The blood samples were harvested from the spinal cord I/R and sham rabbits at 48 h after operation. The differences between two groups were analyzed using by the Student's *t*-test. Bars represent mean ± SD (*n* = 6–8). ^*^P < 0.05 vs. the Sham group.

**Figure 2:**
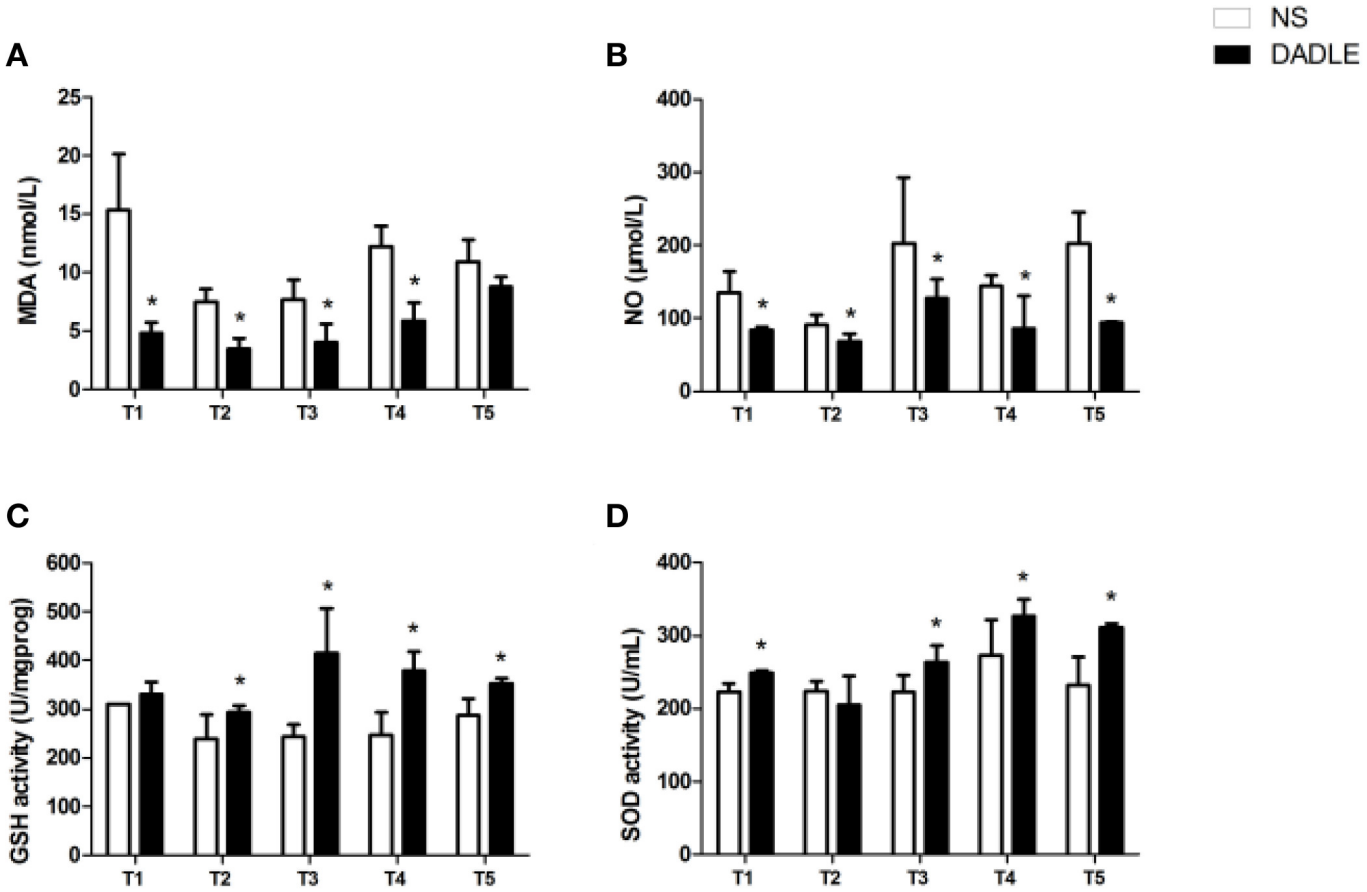
DADLE-induced decrease in MDA and NO levels and increase in GSH-Px and SOD levels in spinal cord I/R injury. **(A)** The effects of DADLE on MDA activity. **(B)** The effects of DADLE on NO activity. **(C)** The effects of DADLE on GSH-Px activity. **(D)** The effects of DADLE on SOD activity. T1 = the onset of reperfusion; T2 = 1 h after reperfusion; T3 = 6 h after reperfusion; T4 = 24 h after reperfusion; T5 = 48 h after reperfusion. Differences in means in NS and DADLE groups at each time point were analyzed using two-way ANOVAs followed by the Bonferroni *post hoc* test. Bars represent mean ± SD (*n* = 8). ^*^P < 0.05 vs. the NS group.

As reported in Figure 1, serum MDA level in group NS was significantly increased compared with the Sham group at T5 (*P* < 0.05, Figure 1A). In addition, significant differences were presented between the NS and DADLE groups from the time points of T1 to T4. Compared to the NS group, DADLE treatment remarkably decreased MDA activity in the blood (*P* < 0.05, Figure 2A).

### Assessment of the level of serum NO

Accordingly, the data revealed that serum NO from ischemic animals that treated with NS significantly increased compared with the Sham group at T5 in rabbits (*P* < 0.05, Figure 1B). However, compared with the NS group, NO concentration was remarkably attenuated by DADLE treatment at all-time points (*P* < 0.05, Figure 2B).

### Assessment of the level of serum GSH-PX

As shown in Figure 1C, ischemic spinal cords induced a significant reduction of GSH-Px activities compared with the Sham group at T5 (*P* < 0.05). However, our results showed that administration of DADLE significantly reversed such reduction compared with the NS treated group from T2 to T5, especially at T3 (*P* < 0.05, Figure 2C).

### Assessment of the level of serum SOD

The analysis of data revealed that a lower level of SOD was observed in the NS group compared with the Sham group at T5 (Figure 1D). The change of SOD activity was similar to GSH-Px in response to NS and DADLE treatment. SOD level was significantly higher in the DADLE group compared with the NS group at the time points of T1 and from T3 to T5 (*P* < 0.05), and there was no significant change between two groups at T2 (Figure 2D).

### Assessment of the level of caspase-3

**Figure 3:**
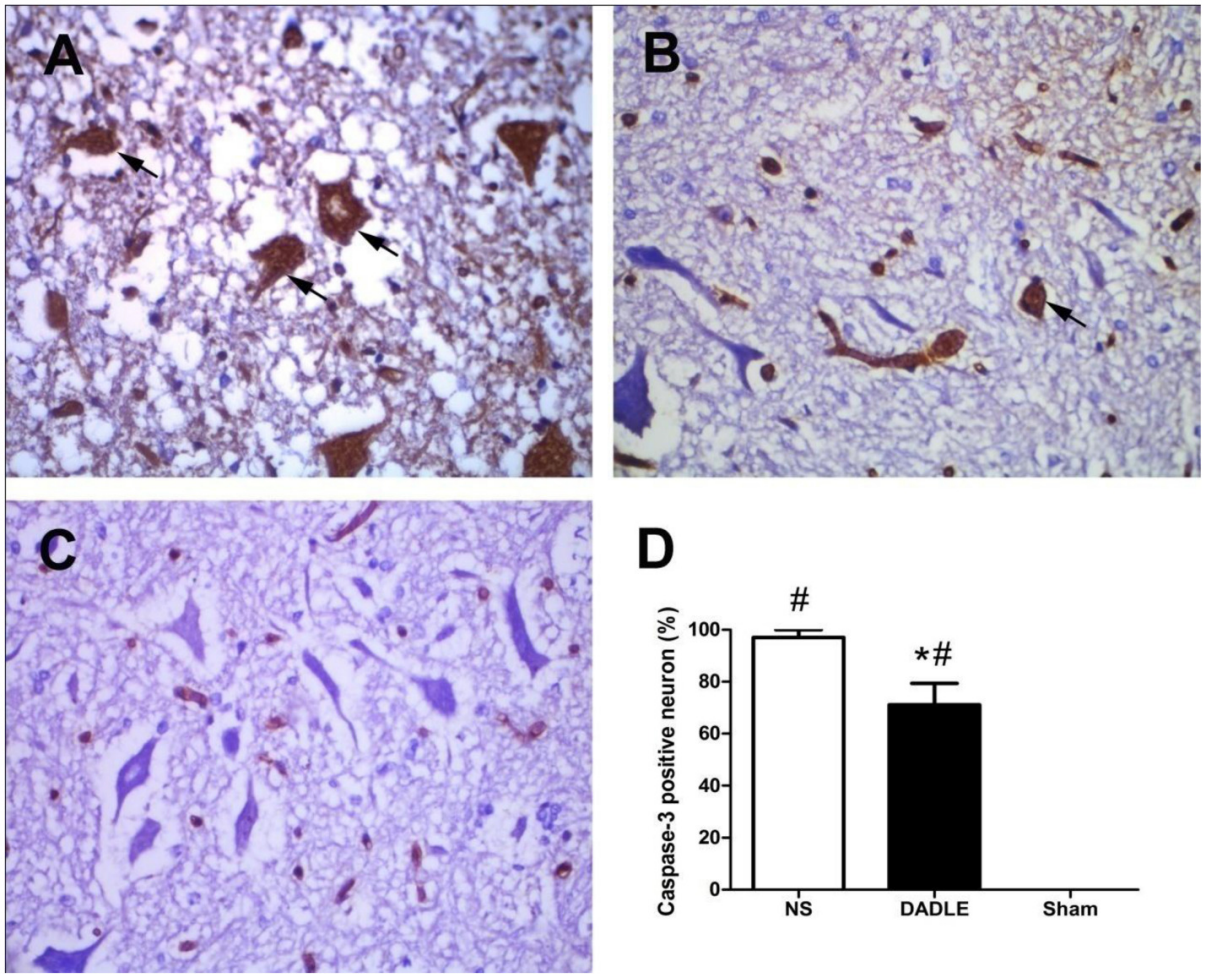
Immunohistochemical staining of spinal cords using caspase-3 antibody at 48 h after reperfusion. The samples of spinal cords were harvested from the spinal cord I/R and sham rabbits at 48 h after operation. Neurons in Group NS contained intense caspase-3 immunoreactivity **(A)**, which was significantly attenuated in Group DADLE **(B)**, while normal spinal cords stained poorly with caspase-3 in Group Sham **(C)**. Comparison of percentage of caspase-3 positive neuron in each high power field **(D)**. Bars represent mean ± SD (*n* = 6–8). ^*^P < 0.05 vs. the NS group. ^#^*P* < 0.05 vs. the Sham group. The arrows indicate caspase-3 positive cells.

To evaluate whether DADLE reduced caspase activity in the ischemic spinal cord, caspase-3 expression was examined using immunohistochemical methods. The results showed that caspase-3 immunoreactivity was barely detectable above background in the Sham group, while there was intense protein immunoreactivity in saline-treated ischemic animals. Moreover, DADLE treatment significantly decreased caspase-3 positive cells (*P* < 0.05, Figure 3). These results indicated that DADLE could protect the spinal cord against apoptotic cell death process associated with I/R injury.

### Assessment of the level of p53 expression

**Figure 4:**
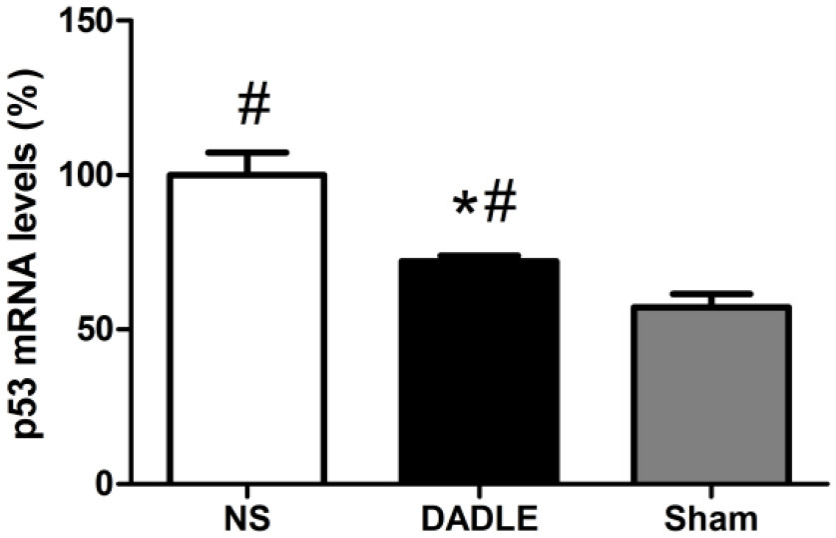
Quantitative p53 mRNA expression through real-time PCR in the spinal cords at 48 h after reperfusion. The samples of spinal cords were harvested from the spinal cord I/R and sham rabbits at 48 h after operation. The transcript level of p53 were up-regulated in the normal saline-treated rabbits, while a marked reduction was shown in the DADLE group. Bars represent mean ± SD (*n* = 6–8). ^*^P < 0.05 vs. the NS group. ^#^*P* < 0.05 vs. the Sham group.

We performed real-time PCR and western blot to evaluate whether DADLE affected the expression of p53 at 48 h after spinal cord I/R. The real-time PCR analysis showed a significant increase of p53 mRNA expression in saline-treated rabbits at 48 h after reperfusion compared with the Sham group (*P* < 0.05), while this increase was attenuated by DADLE (*P* < 0.05, Figure 4).

**Figure 5:**
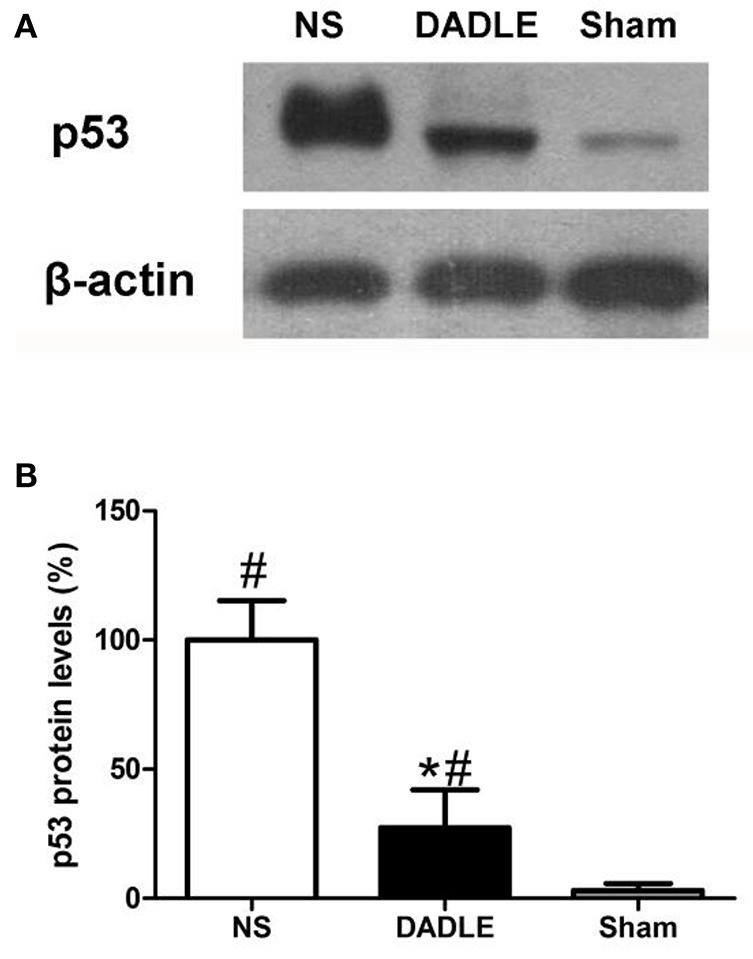
Representative western blot of p53 **(A)** and quantitative analysis of p53 protein **(B)** in the ischemic spinal cords at 48 h after reperfusion. The samples of spinal cords were harvested from the spinal cord I/R and sham rabbits at 48 h after operation. The expression of p53 protein was up-regulated in the NS group, which was significantly decreased by DADLE treatment. Bars represent mean ± SD (*n* = 6-8). ^*^P < 0.05 vs. the NS group. ^#^*P* < 0.05 vs. the Sham group.

The trend is also confirmed at protein level by western blot experiments (Figure 5). The results demonstrated that p53 protein level in the NS group had a significant increase compared with the Sham group at 48 h after reperfusion, which was remarkably reduced by DADLE (*P* < 0.05). These data can be visualized in the representative blot and the thickness of the bands reflects the changes as described above.

## Discussion

Spinal cord I/R injury comprises the most important complication of thoracic and thoracoabdominal aortic aneurysm repair surgery and is considered to be one of the major cause of neurological disability worldwidely. Despite intensive efforts to develop new neuroprotective agents for experimental central nervous system ischemia models, most so far have failed to show clinical effects. Our previous studies showed that DADLE could attenuate spinal cord I/R injury during the ischemic period and the neuroprotective effect appeared most significant at a dose of 0.05 mg/kg (Liu et al., 2016). Antioxidative and antiapoptotic properties of DADLE were only reported *in vitro* experiments or in the model of cerebral I/R injury (Tsao et al., 1998; Hayashi et al., 2002). However, the pathophysiology processes involving the I/R injury in the brain and spinal cord are not identical. In this study, we further investigated whether DADLE protected spinal cord against I/R injury through its antioxidant and antiapoptotic properties on the basis of previous studies.

Two major findings were made in this study. Firstly, our data showed that DADLE attenuated the production of MDA and NO, meanwhile increased the activities of SOD and GSH-Px, indicating that the neuroprotective benefits of DADLE are probably related to antioxidant capacity. Secondly, we found that DADLE administration inhibited the expressions of caspase-3 and p53 which are highly related to apoptosis, suggesting that one protective mechanism of DADLE on spinal cord I/R injury is related to its antiapoptotic property. So we suggest that the delta-opioid peptide DADLE increases the survival of spinal cord exposed to I/R injury via at least two mechanisms: antioxidative and antiapoptotic pathway.

Among the proposed mechanisms involved in spinal cord I/R damage, oxidative stress caused by reactive oxygen species (ROS) including superoxide, hydrogen peroxide, hydroxyl and reactive nitrogen species (RNS) including NO contribute to the pathological process of secondary neuron injury. Under physiological condition, superoxide accumulation was scavenged by the antioxidant enzymes such as GSH-Px and SOD. However, overproduction of oxidants following tissue hypoxia and/or ischemia overwhelm the antioxidant capacities of the cells, resulting the pathogenic outcome of the cell death (Lièvre et al., 2000). The spinal cords mostly consist of lipids and particularly susceptible to peroxidation induced by free radical. Peroxidation of lipids may leading to respiratory dysfunction, mitochondrial permeability transition and consequently cause cell death (Hall et al., 2016). MDA, the production of damaged polyunsaturated fatty acids, generates DNA-protein cross-links and is a highly toxic molecule. It serves as a marker for the extent of lipid peroxidation (Del Rio et al., 2005). Tsao et al. (1998) reported that DADLE functioned as a free radical scavenger in alleviating the generation of superoxide anions, hydroxyl free radicals and lipid peroxidation *in vitro*. In this study, DADLE has been found to share similar mechanism underlying neuroprotection in the ischemic spinal cords. Our current results show that ischemic spinal cords damage increased the serum concentration of MDA compared with the Sham group, which was mitigated by DADLE treatment. In addition, NO as a kind of oxidative stress biomarker has been shown to exert pivotal role in organ I/R injury. Higher NO concentration mediated inflammatory processes following I/R, depressed mitochondrial function and even triggered cellular death (Schulz et al., 2004). In our study, NO was upregulated in the spinal cords after I/R injury, while DADLE partially reversed it.

GSH-Px is the most important antioxidant enzyme by modulating the redox status of proteins in the cell membrane (Zhang et al., 2014). In the present study, we investigated the antioxidant capacity of DADLE by measuring the GSH-Px activity in the blood of rabbits exposed the spinal cord I/R. Consequently, spinal cord I/R injury tended to decrease the GSH-Px level, while DADLE intervention restored it. SOD is another critical antioxidant enzyme that scavenges free radicals generated by the electron transport chain to form H_2_O_2_ and oxygen (Wang et al., 2009). Consistent with GSH-Px, our data demonstrated that serum SOD activity in rabbits of spinal cords exposed to ischemic insult was reduced, which was reversed by DADLE treatment. Our results are consistent with the rat model of cerebral I/R injury, in which DADLE increased the activity of GSH-Px and SOD, and decreased the MDA and NO levels (Yang et al., 2009). Therefore, our results suggest that DADLE may protect spinal cord exposed to I/R insult through antioxidant functions.

Apoptosis plays a prominent role in the pathophysiology of secondary injury to ischemic spinal cord. This harmful process lasts for a long time and results motor neuron death, even if blood flow would be restored later (Hayashi et al., 1998). During this procedure, caspase-3 activation was essential for the execution step in programmed cell death and was a crucial mediator of ischemic injury (MacCorkle et al., 1998). It has been proven that inhibition of apoptosis may be a promising approach for therapeutic intervention in spinal cord injury (Ali-Khan and Hales, 2003). In addition, Hayashi et al. (2002) reported that DADLE showed potent antiapoptotic effects in serum-deprived pheochromocytoma cells. Zheng et al. (2012) suggested that DADLE could protect the brain from I/R injury most likely through the antiapoptotic pathway.

In our present study, we further tried to find that weather DADLE by regional reperfusion into abdominal aorta could inhibit the apoptosis of spinal cord motor neurons. Firstly, we examined if DADLE could affect caspase-3 expression in a rabbit model of spinal cord I/R injury. The results showed higher proportion positive cells exposed to I/R injury compared with the Sham group, indicating the importance of caspase-3 to activation of apoptotic process in spinal cord ischemia. Unsurprisingly, DADLE reduced the caspase-3 expression, suggesting that blocking caspase-3 activation may be a possible mechanism involved in DADLE neuroprotection.

Next, we further investigated the levels of p53 mRNA and protein to evaluate whether DADLE could alter the expression. Because p53 is an associated marker for apoptosis and the suppression of p53 was reported to protect neurons exposed to ischemia insults (Li et al., 1994). Consequently, we found that p53 expression increased in accordance with the result of caspase-3, meanwhile DADLE mitigated these changes, which suggested that DADLE limited neuron apoptosis possibly via inhibiting p53 signaling pathway.

The current study has several limitations. The specific site of action of DADLE remains unknown. However, the major goal of the current study was to investigate the effects of DADLE on oxidative stress and apoptosis. The future studies should include the investigation of whether DADLE provides neuroprotection in spinal cords at a delta-opioid receptor dependent-way or a delta-opioid receptor independent-way. Secondly, we did not assess the tolerance to DADLE upon long-term treatment in the spinal cord tissue. It is still an important area which we will investigate in the future studies.

In conclusion, our data demonstrated that DADLE can protect spinal cord neurons in the anterior horn exposed to I/R damage by decreasing MDA and NO levels and enhancing post-ischemic GSH-Px and SOD levels, and also by inhibiting apoptosis through a mechanism associated with caspase-3 and p53. These findings suggest that DADLE will be a promising therapeutic agent in the spinal cord I/R injury induced by aorta occlusion.

## Author contributions

JY designed the study. DF and HL performed the experimental phase. DF and HL collected and analyzed data. DF drafted the manuscript. SL and LC contributed with reagents/materials/analysis tools. DF, HL, SL, LC, and JY have collaborated and approved the final manuscript version.

### Conflict of interest statement

The authors declare that the research was conducted in the absence of any commercial or financial relationships that could be construed as a potential conflict of interest.

## Abbreviations

[D-Ala^2^, D-Leu^5^]: DADLE
I/R: ischemia-reperfusion
MDA: malondialdehyde
NO: nitric oxide
GSH-Px: glutathione peroxidase
SOD: superoxide dismutase
NS: normal saline
PCR: polymerase chain reaction.
